# Association between county-level sociodemographic characteristics and county-level differences in opioid dispensing

**DOI:** 10.1016/j.pmedr.2021.101612

**Published:** 2021-10-25

**Authors:** Laura J. Cremer, Natasha Underwood, Amber Robinson, Gery P. Guy, Cherie R. Rooks-Peck

**Affiliations:** Division of Overdose Prevention, National Center for Injury Prevention and Control, Centers for Disease Control and Prevention, Atlanta, GA, USA; a This project was supported in part by an appointment to the Research Participation Program at the Centers for Disease Control and Prevention administered by the Oak Ridge Institute for Science and Education through an interagency agreement between the US Department of Energy and the Centers for Disease Control and Prevention.

**Keywords:** Prescription opioids, Health equity, Social determinants of health

## Abstract

**Background:**

While overall opioid prescribing has been decreasing in the United States, the rates of prescribing at the county level have been variable. Previous studies show that social determinants of health (the social and economic conditions in which we live) may play a role in opioid prescribing; however, researchers have not examined this relationship across US counties. This cross-sectional study seeks to determine whether county-level sociodemographic characteristics (e.g., economic, housing, social environment, healthcare environment, and population characteristics) are associated with county level differences in opioid dispensing.

**Methods:**

Data from 2,881 counties in the United States from 2017 to 2018 were used for this study. Opioid dispensing was measured using morphine milligram equivalents (MME) per capita. Spatial error models were used to measure the association between county-level sociodemographic characteristics and MME per capita while adjusting for spatial correlation between neighboring counties.

**Results:**

In the adjusted model, counties with a higher percentage of people below the poverty line, with less than a 4-year college degree, and without health insurance were associated with higher MME dispensed per capita, as were counties with higher percentages of families headed by a single parent, persons separated or divorced, and those with disabilities. Conversely, minority race/ethnicity and rural population were associated with lower opioid dispensing.

**Conclusions:**

County-level sociodemographics can differ in their association with opioid dispensing, hence examining which county-level factors help in improving opioid prescribing, and implementing overdose prevention strategies that tackle these factors is important.

## 1 Introduction

The amount of opioids prescribed in the United States has been decreasing since 2012 (Guy et al., 2017). Although numbers declined overall, opioid prescribing at the county-level has varied, with some observing increasing amounts in recent years (Guy et al., 2017). Identifying factors contributing to differences in county-level prescribing is important for overdose prevention. Previous studies (Barocas et al., 2019, Han et al., 2012) show social determinants of health (SDOH) play a role in opioid prescribing-related outcomes (e.g., use of multiple prescribers and pharmacies). One study showed individuals who resided in counties where <4% of the population belonged to multiple ethnicities had approximately 5% lower incidence rates of new prescriber use of opioids and 8% higher incidence rates in counties where 30% or more residents did not graduate from high school (Han et al., 2012). However no studies have examined the relationship between SDOH and opioid prescribing with a national perspective. SDOH are conditions or factors in the environment in which people live that impact their health and well-being (Adler and Stewart, 2010, Wilkinson and Marmot, 2003). Economics, housing, social environment, healthcare environment, and population characteristics capture county-level sociodemographics and are informed by WHO’s SDOH (Solar and Irwin, 2010) and socioecological frameworks (Macintyre et al., 2002). These frameworks encouraged researchers to study relationships between county-level sociodemographic characteristics and drug-related mortality (Monnat, 2018, Monnat et al., 2019). Although opioid prescribing rates were related to overdose mortality (Monnat et al., 2019), the relationship between opioid prescribing and county-level sociodemographic factors has not been examined. This analysis determines whether county-level sociodemographic characteristics are associated with county-level differences in opioid dispensing across the United States. Understanding these relationships can better inform future opioid prescribing and overdose prevention initiatives.

## 2 Methods

### 2.1 Description of outcome

This cross-sectional study examined associations between county-level sociodemographic characteristics and opioid dispensing. We obtained opioid dispensing data from a retail pharmacy dataset (IQVIA Xponent), which includes weighted estimates of prescriptions dispensed from 50,400 retail pharmacies, covering roughly 92% of dispensed prescriptions in the United States. We measured county-level opioid dispensing in 2018 as morphine milligram equivalents (MME) per capita which was calculated from the total MME (sum of MME across all prescriptions) divided by the population (Centers for Disease Control and Prevention, Guy et al., 2017) for each county. In 2018, opioids were dispensed in 2,881 of the 3,242 counties (91%) (including 135 county equivalents (e.g., District of Columbia or Louisiana Parishes)) in the United States and its territories.

### 2.2 Measures

County-level sociodemographic characteristics included economics, housing, social environment, healthcare environment, and population and were informed by WHO’s SDOH (Solar and Irwin, 2010) and socioecological frameworks (US Department of Health Human Services, 2020), and potential associations with county-level differences in opioid-related mortality (Monnat, 2018, Monnat et al., 2019). We obtained county-level sociodemographic factors from the US Census Bureau. The percentage of rural population in a county is from 2010, the most recent year available. All other county-level variables were from 2017. Economic characteristics included percentages of the population below the poverty line (ages 18–64 years), unemployed (ages 20–64 years), less than a 4-year college degree (ages 25–64 years), and without health insurance (ages 19–64 years). We examined percentages of vacant housing units and renter-occupied housing units with rent<30% of household income as housing characteristics (Monnat, 2018). Social environment characteristics included percentages of families with children headed by a single parent and of persons separated or divorced (older than age 15 years). We included physicians and surgeons per 10,000 persons to assess healthcare environment characteristics. Population characteristics included median age, percentages of those who identified as Black, American Indian and Alaska Native (AI/AN), Hispanic, have a disability, and percentage of the rural population. Areas identified as “urban” contained at least 2,500 persons. (U.S. Census Bureau, 2019). All other areas are considered “rural”.

### 2.3 Statistical analyses

We used spatial error models (SEM) to examine associations between measures of SDOH and MME per capita at the county level. We conducted a Moran’s I test with a spatial weight matrix based on first order queen contiguity to test for spatial autocorrelation (clustering of values across geographic spaces). This categorized any counties sharing a border as neighbors. Neighboring counties influence one another and a positive Moran’s I showed (I = 0.20, p < 0.001) they are more similar than non-neighboring counties and the need for a model accounting for counties’ geospatial location. Lagrange Multiplier test showed an SEM would best represent the spatial autocorrelation, which explains spatial correlation in the error term of the model. We used two analytic models to examine the relationship between sociodemographic characteristics and opioid dispensing. Bivariate SEMs examined predictor variables and MME per capita. Pearson’s correlation coefficients and variance inflation factor test determined predictor variables were not highly correlated (Variance Inflation Factor < 5.0) (Craney and Surles, 2002, Vatcheva et al., 2016); therefore, all variables were included in the multivariable model. We then examined associations between all SDOH predictor variables and MME per capita using a multivariable SEM.

We used function errorsarlm in package spdep with R version 3.6.1 for analyses. This study was exempt from human-subject regulations and institutional review board approval.

## 3 Results

### 3.1 Preliminary analyses

The average amount of opioids dispensed in 2018 was 500.7 MME per capita and ranged from 0.1 to 2,718.7 MME per capita (Table 1).

**Table 1:** Descriptive Statistics for morphine milligram equivalents (MME) dispensed per capita and social determinants of health at the county level.

Variable^a^	Mean (SD)	Range
MME per capita (2018)^b^	500.66 (304.61)	0.1 – 2718.7
Economic characteristics		
Population below the poverty line, ages 18–64 years, %	15.51 (6.15)	2.80 – 45.00
Civilian non-institutionalized population unemployed or not in labor, ages 20–64 years, %	68.69 (9.41)	11.20 – 93.0
Population with < 4-year college degree, ages 25–64 years, %	77.98 (10.23)	20.45 – 95.42
Population without health insurance, ages 19–64 years, %	15.96 (7.02)	2.72 – 56.47
Housing characteristics		
Vacant housing units, %	17.62 (10.23)	3.05 – 82.11
Renter-occupied housing units with rent ≥ 30% of housing income, %	46.47 (8.06)	9.80 – 76.10
Social Environment characteristics		
Families with children headed by single parent, %	15.95 (4.62)	2.79 – 43.11
Persons separated/divorced, ages ≥ 15 years, %	13.63 (2.58)	3.80 – 27.80
Healthcare environment characteristics		
Health diagnosing and treating practitioners per 10,000 population	157.15 (63.09)	0.0 – 718.66
Population characteristics		
Median age	40.89 (5.08)	21.60 – 66.40
Black population, %	9.40 (14.53)	0.00 – 86.92
Hispanic population, %	8.89 (13.30)	0.00 – 99.19
American Indian and Alaska Native population, %	1.50 (5.29)	0.00 – 78.22
Rural population,^c^ % (2010)	55.63 (30.65)	0.00 – 100.00
Civilian non-institutionalized population with disability, %	15.90 (4.36)	4.50 – 34.20

a Sociodemographic characteristics were collected from the 2017 American Community Survey.

b MME per capita is calculated based on 2018 dispensing data from IQVIA Xponent.

c Percentage of rural population was collected from the 2010 Census because it was the most recent data available.

### 3.2 Bivariate analyses

Table 2 displays bivariate and multivariable regression estimates and confidence intervals (CI) for SEMs of MME dispensed per capita. The bivariate analysis showed significant predictors of higher opioid dispensing within a county. These included higher percentages of the population below the poverty line, having less than a 4-year college degree, families with children headed by a single parent, being divorced or separated, having a disability, and higher ratios of physicians to a population. Increasing unemployment, vacant housing units, rural population, Hispanic, and AI/ANs were associated with lower opioid dispensing.

**Table 2:** Bivariate and Multivariable Spatial Error Regression of morphine milligram equivalents (MME) dispensed per capita associated with social determinants of health.

	MME per capita^b^ Estimate (95% CI)	
Variable^a^	Bivariate	Multivariable^c^
Economic characteristics		
Population below the poverty line, ages 18–64 years, %	**7.19 (5.20, 9.18)**	**5.76 (2.90, 8.62)**
Civilian non-institutionalized population unemployed or not in labor, age 20–64 years, %	**−2.59 (−3.98, −1.20)**	0.95 (**−**0.98, 2.88)
Population with < 4-year college degree, ages 25–64 years, %	**2.05 (0.85, 3.26)**	**3.88 (2.06, 5.70)**
Population without health insurance, ages 19–64 years, %	0.72 (**−**1.27, 2.71)	**3.93 (1.51, 6.35)**
Housing characteristics		
Vacant housing units, %	**−1.77 (−2.95, −0.59)**	0.91 (**−**0.50, 2.32)
Renter-occupied housing units with rent ≥ 30% of housing income, %	−0.69 (**−**2.16, 0.78)	1.59 (**−**0.03, 3.21)
Social Environment characteristics		
Families with children headed by single parent, %	**9.55 (6.82, 12.26)**	**7.07 (2.73, 11.41)**
Persons separated/divorced, ages ≥ 15 years, %	2**7.97 (23.56, 32.39)**	**6.21 (0.77, 11.64)**
Healthcare environment characteristics		
Health diagnosing and treating practitioners per 10,000 population	**0.19 (0.02, 0.36)**	0.17 (**−**0.03, 0.38)
Population characteristics		
Median age	0.30 (**−**1.90, 2.51)	2.44 (**−**0.76, 5.65)
Black population, %	0.45 (**−**0.61, 1.50)	**−4.94 (−6.27, −3.60)**
Hispanic population, %	**−2.58 (−3.75, −1.41)**	**−6.90 (−8.23, −5.57)**
American Indian and Alaska Native population, %	**−3.54 (−5.76, −1.33)**	**−6.96 (−9.36, −4.55)**
Rural population,^d^ % (2010)	**−2.55 (−2.91, −2.20)**	**−4.28 (−4.77, −3.79)**
Civilian non-institutionalized population with disability, %	**16.78 (13.91, 19.66)**	**16.16 (11.80, 20.53)**

a Sociodemographic characteristics were collected from the 2017 American Community Survey.

b MME per capita is calculated based on 2018 dispensing data from IQVIA Xponent.

c Multivariable analysis includes all economic, housing, social and healthcare environments, and population characteristics.

d Percentage of rural population was collected from the 2010 Census because it was the most recent data available.

### 3.3 Multivariable analyses

We found significant associations with higher opioid dispensing after including all predictor variables in the model. These include percentages of the population below the poverty line (β = 5.76, 95% CI = 2.90, 8.62), without a 4-year college degree (β = 3.88, 95% CI = 2.06, 5.70), without health insurance (β = 3.93, 95% CI = 1.51, 6.35), families headed by a single parent (β = 7.07, 95% CI = 2.73, 11.41), persons separated or divorced (β = 6.21, 95% CI = 0.77, 11.64), and with a disability (β = 16.16, 95% CI = 11.80, 20.53). Conversely, populations with higher percentages of Blacks (β = −4.94, 95% CI = −6.27, −3.60), Hispanics (β = −6.90, 95% CI = −8.23, −5.57), AI/AN (β = −6.96, 95% CI = −9.36, −4.55), and rural population (β = −4.28, 95% CI = −4.77, −3.79) were associated with lower opioid dispensing.

Percentages of the population without health insurance and Black population were not statistically significant predictors of opioid dispensing in the bivariate model, but statistically significant in the multivariable model. Once all covariates were controlled for in the adjusted model, the association between opioid dispensing and percentage of the population without health insurance was positive while the association between opioid dispensing and percentage of Black population was negative. Housing characteristics were not associated with MME per capita in either analyses.

The positive spatial correlation coefficient was statistically significant in the bivariate and multivariable models (λ = 0.42, p < 0.001) showing the spatial location of counties is a significant predictor of opioid dispensing after considering county sociodemographic characteristics.

## 4 Discussion

This study showed associations between county-level sociodemographic characteristics and county-level opioid dispensing. Lower economic and social environmental status (e.g., percentages of population separated or divorced and families headed by a single parent) and disability were positively associated with opioid dispensing. Conversely, higher percentages of Blacks, Hispanics, and AI/ANs were negatively associated with opioid dispensing. Previous research has shown connections between socioeconomic factors and prescription opioid overdoses (Boslett et al., 2019, Pear et al., 2019a); however, this is one of the first studies assessing these factors and opioid dispensing. Researchers should further investigate relationships between opioid dispensing, overdose deaths, and socioeconomic factors to develop tailored prevention interventions (Cerdá et al., 2017, Pear et al., 2019b). This study showed that across the US, county-level sociodemographic characteristics vary in their relationship with opioid dispensing. In neighboring counties that have similar sociodemographic characteristics (Tabb et al., 2018), regional interventions implemented among county clusters may be beneficial (Wallace et al., 2019). Future studies should identify places by accounting for underlying SDOH, that may benefit from additional efforts to understand, monitor, and improve opioid dispensing.

Prior studies showed individual-level factors like race/ethnicity and health insurance status is related to opioid prescribing (Gaither et al., 2018, Janakiram et al., 2018, Singhal et al., 2016). These studies focused on relationships between individual-level prescribing and individual-level demographics, such as race, within specific settings or populations. A previous analysis showed receipt of an opioid prescription was positively associated with higher proportions of white and lower-income populations (Friedman et al., 2019) while another found county-level opioid dispensing was positively associated with percentages of white non-Hispanic, African American, and poverty rate (McDonald et al., 2012). In this study, higher levels of opioid dispensing were associated with lower percentages of minorities living in a county. These studies show the consistent role of poverty as a predictor of opioid dispensing. Similar to the relationships observed between opioid dispensing and health insurance in this study, previous studies have shown higher county-level rates of uninsured, as well asMedicaid enrollment are both associated with higher amounts of prescribed opioids (Guy et al., 2017). Additionally, this study showed lower rates of opioid prescription dispensing in counties with a higher percent of rural populations. This could indicate inadequate access to opioids in counties with higher percentages of minorities, higher rates of uninsured and more rural counties. Differences in opioid dispensing by county-level sociodemographic characteristics suggest inconsistent prescribing and demonstrate the need for better applications of opioid prescribing guidance and standards. Communities can use these findings to identify high-prescribing areas for interventions such as academic detailing or individual educational visits to clinicians. Varying previous study findings and our analysis show additional research is needed to better understand the role of sociodemographics and their relationship to opioid dispensing. Prescriber behavior may be an important predictor of opioids dispensed; however, examining provider or patient-level factors was beyond the scope of this analysis.

This study had several limitations. This was a cross-sectional study and we could not determine whether SDOH played a causal role in opioid dispensing. Future studies should examine pathways through which SDOH affect opioid dispensing—and how these factors impact behaviors over time. Although county-level sociodemographics from 2017 are assumed to be representative, the most recent data available for the rural population in a county is from 2010. The rural population could have changed significantly and these results may not reflect the association between rural population and opioid dispensing. Additionally, it is possible that patients could receive prescriptions outside of the county in which they reside. Data in this study were at the aggregate level and cannot be used to make inferences about individual-level associations. We were unable to determine the appropriate level of prescribing for a geographic area because we did not have data on diagnoses. More research is needed to better understand these relationships including among county-level sociodemographics, individual-level characteristics, and individual prescribing.

## 5 Conclusions

Although opioid prescribing levels have decreased (Guy et al., 2017), opioid dispensing remains high and varies considerably across the United States. We found county-level factors are associated with variations in opioid dispensing. This study contributes to the body of literature examining provider dispensing behavior and expands our understanding of it across US counties. As we continue to address the opioid overdose epidemic, strategies focused on prescribing should be effective for all impacted populations and incorporate contextual and community factors such as social determinants of health.

## 6 Disclaimer

The findings and conclusions in this report are those of the authors and do not necessarily represent the official position of the Centers for Disease Control and Prevention.

### CRediT authorship contribution statement

**Laura J. Cremer:** Conceptualization, Methodology. **Natasha Underwood:** Conceptualization. **Amber Robinson:** Conceptualization. **Gery P. Guy:** Conceptualization, Data curation. **Cherie R. Rooks-Peck:** Conceptualization.

## Declaration of Competing Interest

The authors declare that they have no known competing financial interests or personal relationships that could have appeared to influence the work reported in this paper.
